# Understanding the Growth of Electrodeposited PtNi Nanoparticle Films Using Correlated *In Situ* Liquid Cell Transmission Electron Microscopy and Synchrotron Radiation

**DOI:** 10.1021/acs.nanolett.4c02228

**Published:** 2024-08-15

**Authors:** Magdalena Parlinska-Wojtan, Tomasz Roman Tarnawski, Joanna Depciuch, Maria Letizia De Marco, Kamil Sobczak, Krzysztof Matlak, Mirosława Pawlyta, Robin E. Schaeublin, See Wee Chee

**Affiliations:** † Institute of Nuclear Physics Polish Academy of Sciences, PL-31-342 Krakow, Poland; ‡ Department of Biochemistry and Molecular Biology, Medical University of Lublin, Chodzki 1, 20-093 Lublin, Poland; § Department of Interface Science, Fritz-Haber-Institute of the Max-Planck Society, Faradayweg 4-6, 14195 Berlin, Germany; ∥ Faculty of Chemistry, Biological and Chemical Research Centre, 02-089 Warszawa, Poland; ⊥ Solaris National Synchrotron Radiation Centre, Jagiellonian University, Czerwone Maki 98, 30-392 Krakow, Poland; # Materials Research Laboratory, Silesian University of Technology, Konarskiego 18A, 44-100 Gliwice, Poland; 7 ScopeM-Scientific Center for Optical and Electron Microscopy, ETH Zürich, 8093 Zürich, Switzerland

**Keywords:** PtNi nanoparticles, LC-TEM, electrodeposition, STXM, *in situ*

## Abstract

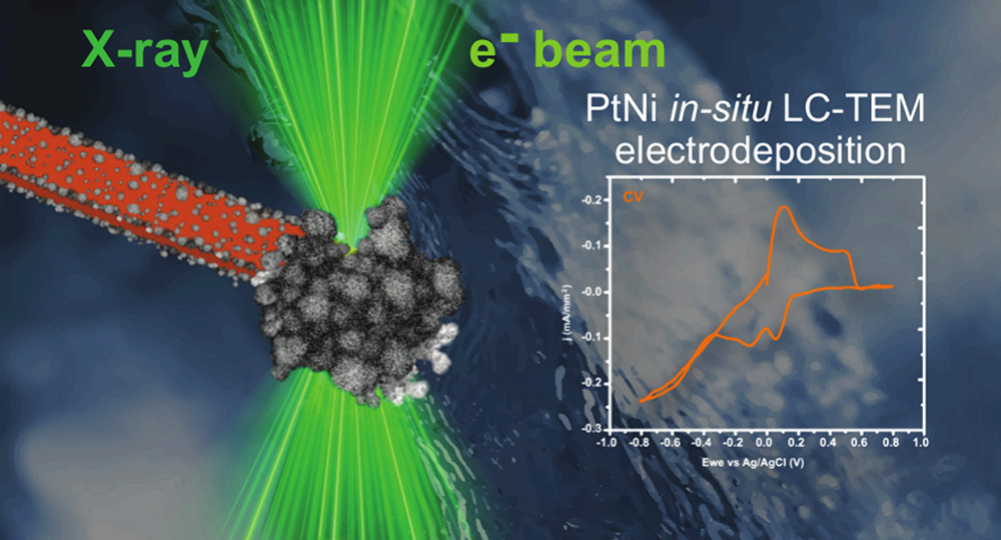

Electrodeposition is a versatile method for synthesizing nanostructured films, but controlling the morphology of films containing two or more elements requires a detailed understanding of the deposition process. We used liquid cell transmission electron microscopy to follow the electrodeposition of PtNi nanoparticle films on a carbon electrode during cyclic voltammetry. These *in situ* observations show that the film thickness increases with each cycle, and by the fourth cycle, branched and porous structures could be deposited. Synchrotron studies using *in situ* transmission X-ray microscopy further revealed that Ni was deposited in the oxide phase. *Ex situ* studies of bulk electrodeposited PtNi nanoparticle films indicated the number of cycles and the scanning rate were the most influential parameters, resulting in a different thickness, a different homogeneity, a different nanoparticle size, and a different surface structure, while the precursor concentration did not have a significant influence. By varying the potential range, we were able to obtain films with different elemental compositions.

Metallic nanoparticles (NPs) are increasingly integral to industrial sectors such as energy, environment, food, and medicine. Electrodeposition is a convenient synthesis approach, allowing the fine-tuning of the particles’ structure under mild aqueous conditions. The particle composition, size, morphology and porosity can be modified by changing parameters such as precursor concentration, potential range, scanning rate, or number of cycles. Furthermore, electrodeposition does not require a specific atmosphere or temperature.^1^ Currently, there are various efforts targeted at using electrodeposition to generate bi- or multimetallic nanostructures.^2^ For example, platinum^3^ and platinum alloyed with other elements such as Ni have been extensively explored for applications in energy and catalysis, due to the ready access of platinum to multiple oxidation states.^4^ In comparison with that of pure Pt, stronger atomic binding between Pt and Ni yields a higher chemical and structural stability of the NPs, resulting in higher mass activity (MA) in electrocatalytic processes, such as the oxygen reduction reaction.^5−7^ A recent density functional theory (DFT) study has shown that a change in the Pt:Ni ratio influences the growth of the PtNi nanocomplex and consequently affects the compressional and shear strain in alloyed PtNi, which, in turn, has an impact on the optimal adsorption of the reaction intermediates and consequently on the efficiency of catalytic reactions.^8^ Strong dealloying at the early stage of cycling is substituted with strong coarsening of catalyst particles at the later stage, which causes a decrease in catalyst efficiency.^9^ Thus, being able to control the morphology and structure of the PtNi NPs is crucial for obtaining materials with improved catalytic properties.^10^

Today, techniques such as scanning electron microscopy (SEM) and transmission electron microscopy (TEM) are commonly used to obtain information about the morphology of the nanostructures post-mortem, but they provide no direct insight into the growth or degradation dynamics, which is needed for tailoring the deposition parameters or understanding the stability of nanostructures. This need for real-time observations of nanostructure evolution has led to several recent advances for *in situ* TEM. In particular, the development of liquid cell TEM (LC-TEM) enabled the visualization of chemical processes in liquid, making it one of the most powerful techniques for elucidating the reaction dynamics in synthesis or dissolution processes.^11,12^ In addition, the integration of electrodes into these liquid cell systems also allows the evolution of the morphology to be studied as a function of time and applied potential, though so far, such work has been primarily on monometal electrodepositions.^13−15^

While electrodeposition is a widely used process for producing coatings for various applications, gaining precise control over the electrodeposited morphology and composition is not trivial. Hence, we wanted to look at electrodeposition in real time to evaluate, which parameters are crucial for control over the growth process. The electrodeposition of bimetallic structures, on the contrary, presents additional challenges due to the difference in the reduction potentials of the two components and the added possibility of processes, such as galvanic replacement, influencing morphological evolution. Being able to look at electrodeposition in real time will allow us to evaluate, which parameters are crucial for the control of the growth process. In this Letter, we report on the use of LC-TEM to study *in situ* the electrodeposition of PtNi NP films using cyclic voltammetry. With real-time imaging, we track the morphological evolution of the films as a function of potential cycling and gain new insight into the growth mechanism at the early stages. These observations are correlated with synchrotron experiments using *in situ* scanning transmission X-ray microscopy (STXM) to additionally obtain the Ni oxidation state.^16^ The morphologies of *ex situ* grown PtNi films were obtained from SEM, energy dispersive X-ray spectroscopy (EDS), and scanning TEM (STEM).

First, *ex situ* electrodeposition of PtNi NPs on a TEM molybdenum grid with carbon lacey foil was performed, which allowed us to analyze their structure in detail by STEM (Figure 1a–d) and EDS (Figure 1e–g). The deposited particles are spherical, as shown in Figure 1a, but high-magnification imaging shows that they consist of branched structures with smaller agglomerated NPs that are assembled in random directions, as shown in Figure 1b. Therefore, the resulting particles have a highly porous surface with a large surface area. The branches are crystalline, as shown in Figure 1c. According to the electron diffraction pattern, the (111), (200), (220), and (311) crystallographic planes were identified as the cubic (fcc) structure of platinum (Figure 1d). The average lattice parameter for the acquired pattern is equal to 3.90 Å for the TEM sample, so it is slightly lower than the parameter of Pt, which is 3.92 Å; this difference can be proof of nickel oxide in the lattice. EDS analysis showed that platinum and nickel are uniformly distributed in the entire NP volume (Figure 1e,f), but the line scan (Figure 1g) shows an increase in the intensities of the Ni and O signals at the edges, suggesting the presence of a thin nickel oxide shell. For the *ex situ* growth of PtNi NPs on a Mo TEM grid, we used parameters optimized in *ex situ* experiments of PtNi electrodeposition on glassy carbon electrodes investigated by SEM and EDS. The influence of the electrodeposition parameters on the film morphology was investigated by systematically varying parameters such as potential range, scanning rate, number of cycles, and Pt:Ni molar ratio. The Supporting Information contains these parameter values (Table S1), as well as voltammetry curves, the SEM images of the surface morphologies, and the EDS results of the grown PtNi NP films shown in Figures S1–S8. From these experiments, we found that a cycling number of <7 and a scanning rate of 80 mV/s allow us to grow a highly porous, but crack-free, film, which consists of small spherical NPs on a glassy carbon electrode.

**Figure 1:**
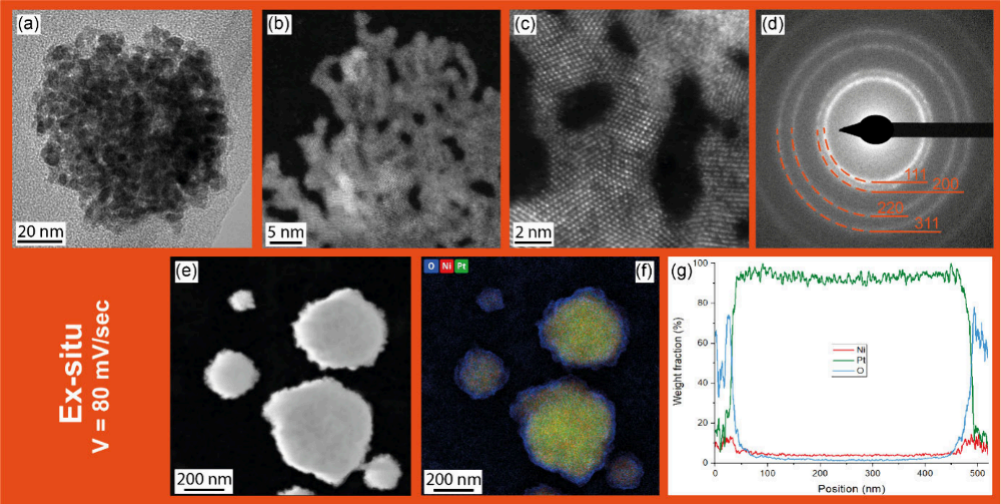
(S)TEM and EDS analysis of the PtNi film grown *ex situ* on a molybdenum TEM grid with lacey carbon foil with the corresponding selected area diffraction pattern. (a) BF TEM overview image of a single nanoparticle. (b) Higher-magnification HAADF STEM image of the branched structure of the NPs. (c) HR-HAADF STEM image of the atomic structure of the branches. (d) SAED corresponding to the particle shown in panel a. (e) HAADF STEM image with (f) corresponding EDS map of the distribution of Pt, Ni and O. (g) Line scan across the bottom right particle.

On the basis of the parameters obtained from the *ex situ* experiments, *in situ* LC-TEM experiments were first performed using a Protochips Poseidon Select and then later in a Hummingbird Scientific liquid cell TEM holder. The second set of LC-TEM experiments was needed for us to directly compare our results with those of the subsequent STXM experiments without additional considerations for the different cell geometries employed by the two vendors. The assembly procedure of the holders is described in the Supporting Information. The preliminary experiments with the Protochips holder are described in the Supporting Information together with a movie of the growth during the first four electrodeposition cycles (Movie 1). Hereafter, we will discuss the results obtained from the Hummingbird Scientific holders.

Figure 2 shows an image sequence extracted from a movie recorded with a magnification of 20000× (Figure 2a) and a frame rate of 1 frame per second (Movie 2), together with the cyclic voltammograms (CVs) obtained from the holder (Figure 2b). The CVs show features that are largely similar to those of CVs obtained from bulk Pt deposition experiments on carbon,^17^ which is not surprising given the high Pt content found in the samples deposited *ex situ*. This agreement allows us to describe the deposition dynamics by correlating the obtained images with the voltammetry data. The first cycle of the electrodeposition is presented as a voltammogram and illustrated by four time frames in Figure S10. Images captured at different times during the first CV show that NP deposition is observed during the first cathodic scan, with a burst in nucleation at −0.2 V (vs Ag/AgCl), and according to the sample contrast, we already have a relatively thick sample after the first cycle. The subsequent panels show additional densification of the film, as indicated by the sample contrast together with a roughening of the deposits on the carbon electrode edge during the second cycle, while we can also see in the CV indicative peaks for Pt and Ni reduction, similar to the results of our *ex situ* experiments (see, for example, Figure S1). Moreover, a comparison of the first cycle and second cycle in the region just before the onset of hydrogen evolution (approximately −0.8 V vs Ag/AgACl) also implies the presence of shallow peaks that are commonly attributed to the adsorption and desorption of hydrogen from Pt,^18^ which would be consistent with the electrodeposition creating new Pt sites for an increased level of hydrogen adsorption.

**Figure 2:**
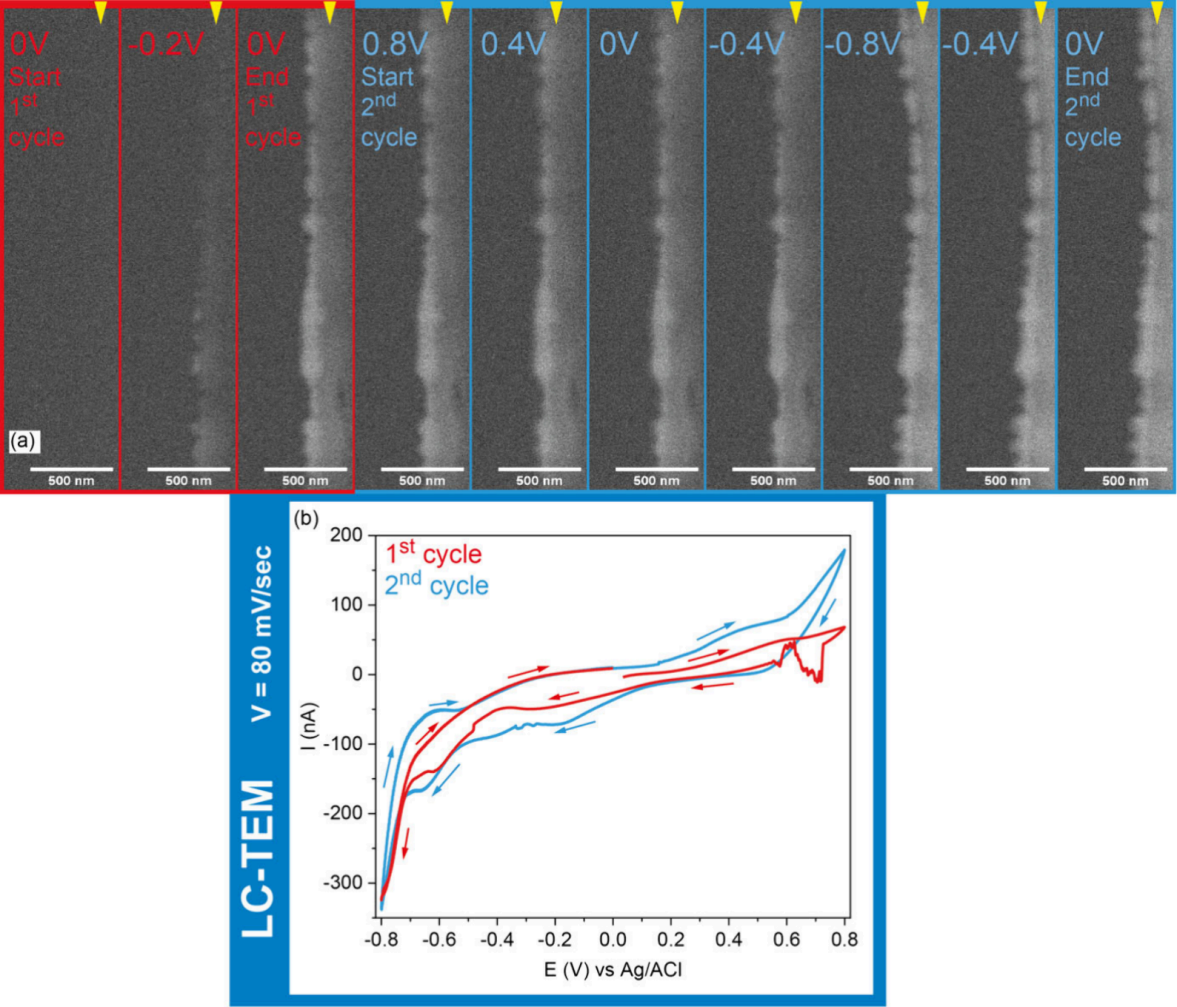
*In situ* LC-TEM image sequence showing the electrodeposition of PtNi on a carbon electrode at low magnification. (a) Frames extracted from Movie 2 at different applied potentials and (b) current–potential curves obtained from scanning the potential between 0.8 and −0.8 V vs Ag/AgCl with a scan rate of 80 mV/s. The electron dose rate was ∼0.7 e^–^ A^–2^ s^–1^. The Hummingbird Scientific holder comes with a customized Ag/AgCl reference electrode built into the holder,^19,20^ and the applied potential is referenced to this internal Ag/AgCl reference. The yellow triangles indicate the electrode edge, which is on the right side of the images.

Next, we performed new experiments with slower frame acquisition (0.35 frame per second) to obtain images with a higher signal:noise ratio and therefore better spatial resolution. Figure 3 shows cropped images extracted from Movie 3, which was recorded at the same scan rate of 80 mV/s, focusing on the growth and dissolution of a few NPs (also provided as Movie 3). Here, we can observe the electrodeposition beginning at approximately −0.8 V versus Ag/AgCl, during the second cycle. When the potential is reversed towards anodic values, the NPs experience partial dissolution, but the electrodeposition is not fully reversed within the potential window between 0.8 and −0.8 V versus Ag/AgCl. Therefore, the particle size oscillates by a few nanometres during each subsequent potential cycle, as one can see in Movies 3 and 4. For instance, between the third and fourth cycles, the lower particle in Figure 3 shrinks by ∼10 nm during the anodic scan and grows by ∼60 nm during the cathodic scan to −0.8 V. However, the particle size steadily increases over the cycles, as one can see by comparing snapshots of particles taken at the same potential over subsequent cycles (Figure 3). More importantly, this increase in size is much more gradual after the fourth CV, leading to the net result that the particles maintain their spherical shape and grow only slowly.

**Figure 3:**
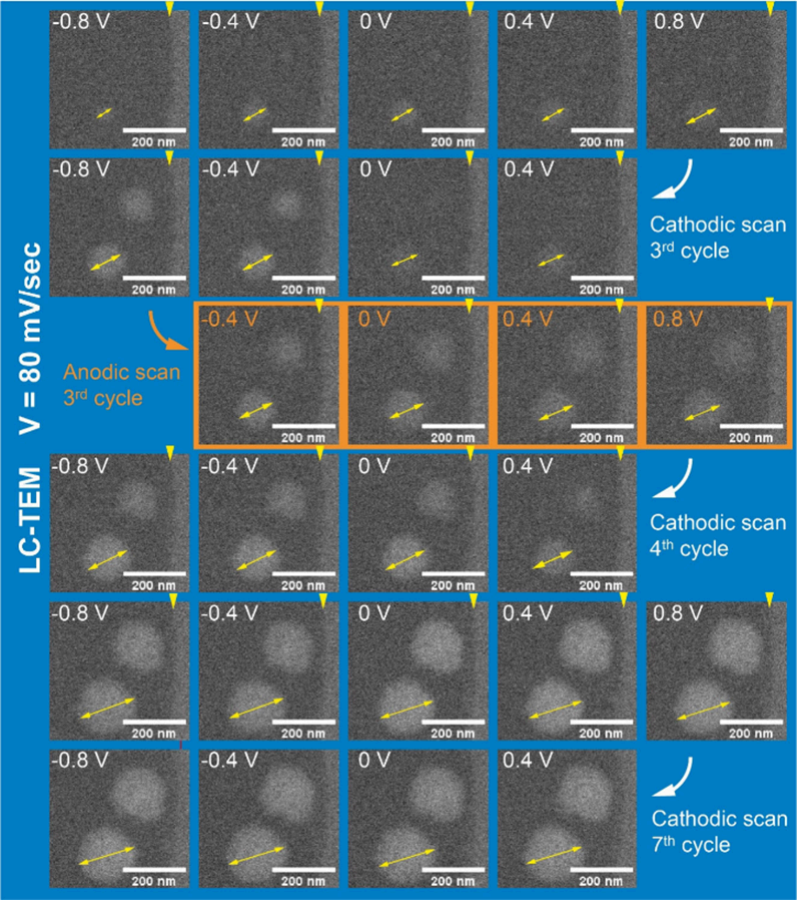
Digital magnification of the nucleation and growth of three particles during the *in situ* electrodeposition of PtNi in LC-TEM, scanning the potential between 0.8 and −0.8 V vs Ag/AgCl with a scan rate of 80 mV/s. Frames were extracted from Movie 3 at different applied potentials during the second to fourth cycles and during the sixth to seventh cycles. The electron dose rate was ∼7 e^–^ Å^–2^ s^–1^. The yellow triangles indicate the electrode edge.

To further investigate the kinetics of the electrodeposition process, we repeated the experiment at a slower scan rate of 10 mV/s as shown in Movie 4. Here, the nucleation of NPs again took place at approximately −0.7 V versus Ag/AgCl during the first cathodic scan, but in contrast to the experiment at 80 mV/s, new NPs continue to be nucleated and existing NP grow as the potential increases from −0.8 to 0 V versus Ag/AgCl. During the first anodic scan, we also see partial dissolution of the deposited particles, as indicated by the lower contrast observed between 0.8 and 0 V versus Ag/AgCl (Figure 4). During the second cathodic scan, the particle size again increases, and the individual particles start to interconnect, leading to the formation of a semicontinuous, porous PtNi film. A substantial increase in particle contrast is observed at approximately −0.8 V during the second cathodic scan, which is concomitant with a higher cathodic current. Unlike the experiment at 80 mV/s, the film deposited at 10 mV/s begins to bend and detaches from the SiN_*x*_ membrane after the second CV (see Movie 4), likely due to stress introduced by the increased amount of deposited material. Nonetheless, the more extensive nucleation and faster growth of the film observed at lower scan rates indicate that the electrodeposition in these experiments is limited by the reaction rate and not by mass transport during the initial cycles.

**Figure 4:**
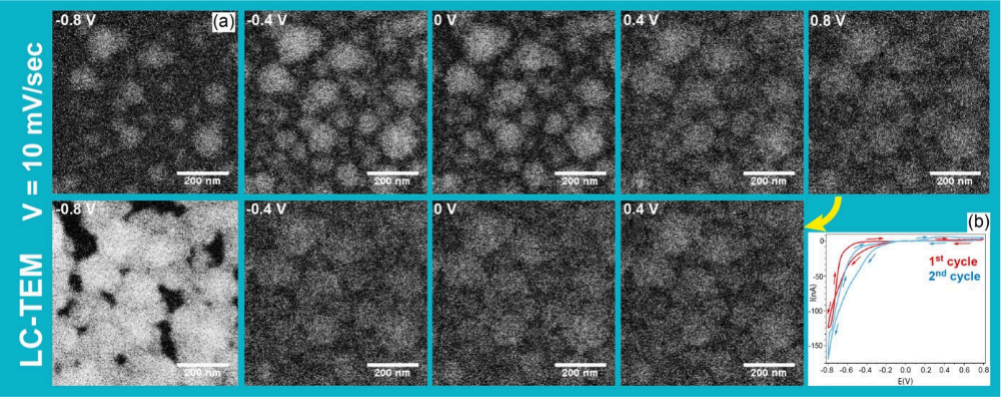
LC-TEM *in situ* electrodeposition of PtNi particles, performed by cycling the potential between 0.8 and −0.8 V vs Ag/AgCl at 10 mV/s. (a) Frames corresponding to different applied potentials, extracted from Movie 4. (b) Current vs potential curves of the first and second cycles of PtNi electrodeposition. The electron dose rate was ∼7 e^–^ Å^–2^ s^–1^.

Next, we performed complementary *in situ* experiments using STXM at the DEMETER beamline at SOLARIS (Krakow, Poland) to verify the concurrent deposition of Ni during the electrodeposition and to obtain its oxidation state using an electrochemical liquid cell holder. In these experiments (Figure 5), the working electrode edge was scanned after every voltammetric cycle at zero potential to investigate the time evolution of the 852.5 eV peak in the Ni adsorption edge, which reflects the Ni^2+^ 2p^6^3d^8^ → 2p^5^3d^9^ transition. This peak (Figure 5b) was visible from the beginning of the experiment, confirming that the nickel that was formed by electrodeposition was in the oxide phase.^16^

**Figure 5:**
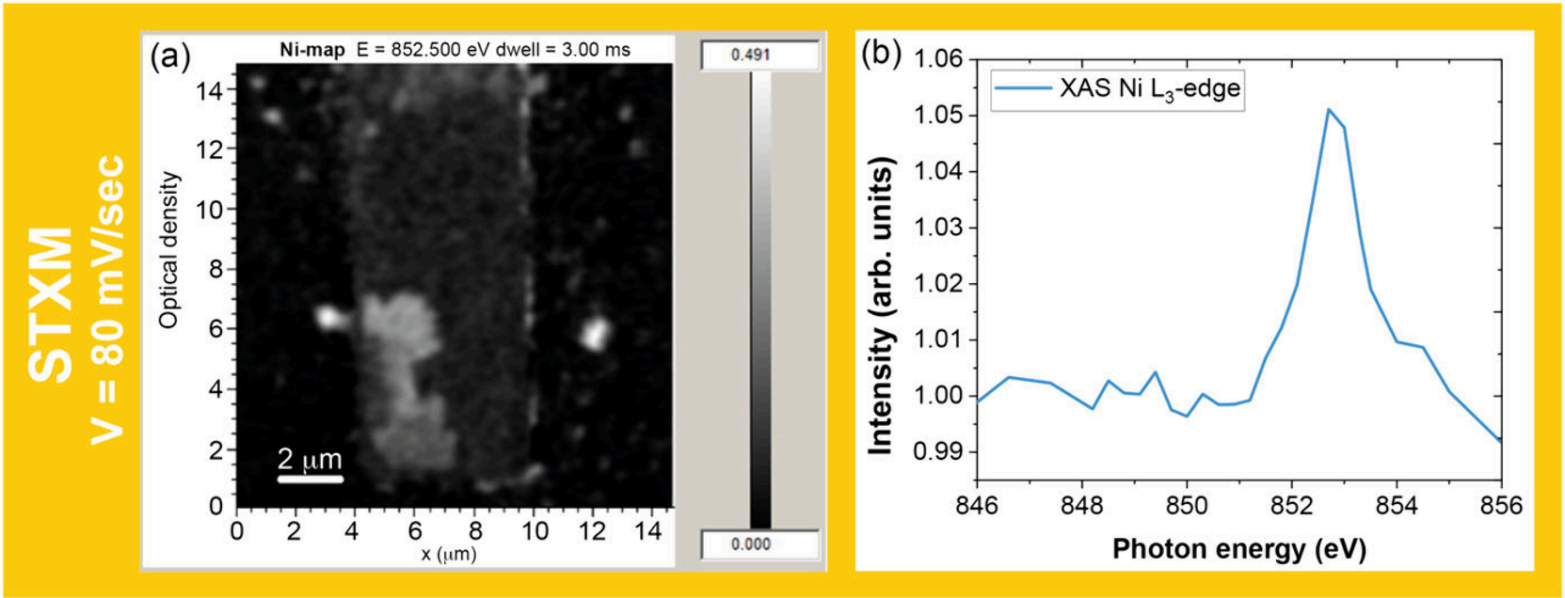
(a) STXM image of the working electrode (dark rectangle) inside the liquid cell placed on a Si_3_N_4_ membrane (dark background). (b) XANES spectra taken from the near edge WE (red box) on the right.

We also performed post-mortem characterization of the films produced during the LC-TEM and STXM experiments and compared them to the samples we prepared *ex situ*, to identify possible discrepancies between the *in situ* and *ex situ* studies. As shown in Figures S14 and S15, all of the electrodeposited PtNi films exhibit a porous, sponge-like structure. However, it was also found from these investigations that the imaged areas showed more deposits and denser films compared to the areas that have not been irradiated by the beam as indicated by panels D and E of Figure S15 (and also Figure S12c), which suggests that the electron beam accelerated the electrodeposition process. The film detachment after the second CV that was seen in Movie 4 also appears to be a consequence of this accelerated film growth due to electron beam imaging.

To further check for other possible undesirable beam-induced artifacts and any discrepancies that may arise for the reaction cell environment (the precursor solution was introduced continuously via liquid flow in the LC-TEM experiments, whereas the *ex situ* experiments are performed in a static solution), we performed post-mortem EDS mapping of the PtNi film electrodeposited *in situ* and *ex situ*. The results are summarized in Table 1 with the obtained maps provided in Figure S15. The quantification reveals that the Pt:Ni elemental atomic ratio for the NP films synthesized *in situ* is in the range between 80:20 and 87:13 found in the sample synthesized with the same synthesis parameters. The sample-to-sample deviations are within the accuracy of EDS quantification, so we can conclude that we are producing NP films with similar composition in both *in situ* and *ex situ* experiments.

**Table 1:**
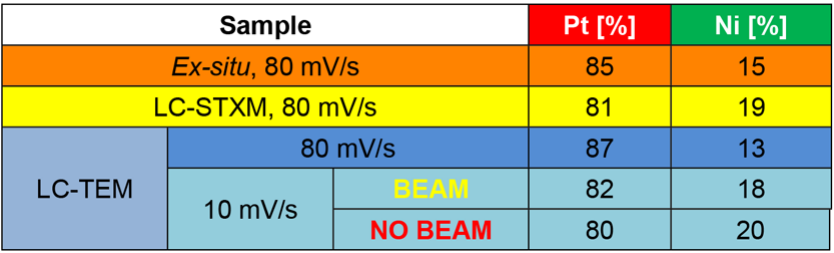
TEM EDS Averaged Quantifications of the Pt and Ni Concentrations in the PtNi Films Grown in the LC-TEM and STXM Experiments

Therefore, the only identifiable effect of the electron beam on our observations is the faster growth rate. This effect may be explained by the electron beam-induced reduction of the precursor ions forming small clusters that act as seeds for the subsequent electrochemical growth and a modification of the growth rate due to the inherent reaction rate-limited kinetics, which we can test by further increasing the magnification (i.e., increasing the electron dose rate). Indeed, we find that at higher magnifications, beam-induced deposition and the emergence of floating NP clusters start to take place, which is shown in Movie 1 and Figure S12c. Nonetheless, we emphasize here that the consistency in morphology and composition between *in situ* and *ex situ* experiments and the dominant effect of the electrodeposition parameters on the growth kinetics strongly suggest that the *in situ* experiments are still representative of real phenomena.

In this work, the electrodeposition of PtNi NP films has been investigated using various parameters and different fabrication environments: *ex situ* and *in situ* in LC-TEM and STXM. First, a series of *ex situ* experiments were performed to optimize the synthesis process to obtain a highly porous surface structure and a nickel concentration of ≥10 atom %. Parameters such as the precursor concentration, voltammetry range, potential scanning rate, and number of voltammetric cycles were varied. With a decrease in the scanning rate and a decrease in the number of cycles, fewer cracks were formed in the NP films composed of spherical nanoseeds. Subsequently, the PtNi NP films were electrodeposited *in situ* during TEM and STXM using electrochemical liquid cell holders for further study. This *in situ* investigation shows that electrodeposition starts at approximately −0.7 V during the first cathodic scan and that the size of the electrodeposited particles oscillates, decreasing during anodic scans and increasing during cathodic scans. Over many scans, the overall particle size increases in a controlled way, and a porous PtNi film is formed, as individual particles interconnect with each other. These real-time observations showed that the synthesis proceeds rapidly in the initial cycles; however, afterward, the dynamics slows and further nanostructure growth is strongly inhibited, which leads to the conclusion that the electrodeposition in these experiments is limited by the reaction rate and not by mass transport during the initial cycles. While the overall compositions obtained from the EDX maps are comparable across the different experiments, a detailed analysis of the local composition reveals significant heterogeneity, with large fluctuations in the Pt content, Table S5 & S6 and Figure S16 & S17. Indeed, the hexachloroplatinic acid in aqueous chloride solutions consists of the PtCl_6_^2–^, PtCl_5_(H_2_O)^−^, and PtCl_5_(H_2_O)_2_ species,^21−25^ and their concentration depends on the pH and temperature of the solution. Due to radiolysis occurring during LC-TEM, the pH of the solution changes locally, inducing variations of the species concentration mentioned above. As these species have different reduction potentials, local variations in the Pt content in our electrodeposited layer are observed. Moreover, at lower scan rates (10 mV/s), a denser film is formed. However, densification of the film eventually leads to delamination due to greater stresses in the film or formation of gas bubbles between the film and the electrode.

To summarize, we have successfully transferred the parameters for PtNi *ex situ* electrodeposition into *in situ* TEM and STXM imaging and recorded real-time observations of the electrodeposition process using both electron and synchrotron beams. These observations show that electrodeposition is limited by the reaction rate and not by mass transport during the initial cycles and allow us to rationalize that the number of cycles is the dominant parameter, as the film growth rate is the fastest during the first four cycles. The Ni is distributed uniformly in the film, while fluctuations in the Pt content are observed. These insights have valuable implications for practical electrodeposition. In addition, we demonstrate how complementary *in situ* scanning X-ray microscopy studies performed using the same liquid cells can provide vital information about the oxidation state of the electrodeposited particles.

## Supporting Information Available

*In situ* TEM movie showing the growth of the PtNi film during electrodeposition (experiment in the Poseidon sample holder, first to fourth cycles, speed × 4) (Movie 1) (MP4)
*In situ* TEM movie showing the growth of the PtNi film during electrodeposition (experiment in the Hummingbird sample holder, 80 mV/s, first and second cycles, movie acquisition speed of 1 frame per second, movie play rate of 5 frames per second) (Movie 2) (AVI)
*In situ* TEM movie showing the growth of the PtNi film during electrodeposition (experiment in the Hummingbird sample holder, 80 mV/s, first and second cycles, movie acquisition speed of 0.35 frame per second, movie play rate of 5 frames per second) (Movie 3) (AVI)
*In situ* TEM movie showing the growth of the PtNi film during electrodeposition (experiment in the Hummingbird sample holder, 10 mV/s, first and second cycles, movie acquisition speed of 0.35 frame per second, movie play rate of 5 frames per second) (Movie 4) (MP4)
*In situ* TEM movie showing the growth of the PtNi film during electrodeposition (experiment in the Poseidon sample holder, destruction of the PtNi NPs by the beam during EDS acquisition) (Movie 5) (MP4)
*In situ* TEM movie showing the growth of the PtNi film during electrodeposition (experiment in the Poseidon sample holder, attachment of the particle to the film) (Movie 6) (MP4)
Details of the electrodeposition of PtNi films, SEM results for the obtained differently structured films, assembly of the liquid cell, and Figures S1–S17 (PDF)

## Supplementary Material

nl4c02228_si_001.mp4

nl4c02228_si_002.avi

nl4c02228_si_003.avi
