# Supplementary material for: Understanding the Growth of Electrodeposited PtNi Nanoparticle Films Using Correlated In Situ Liquid Cell Transmission Electron Microscopy and Synchrotron Radiation
Source: Nano Lett. 2024 Aug 15;24(40):12361–7. doi: 10.1021/acs.nanolett.4c02228 (PMC11468670; doi:10.1021/acs.nanolett.4c02228)
Supplement: Supplementary file 7 — nl4c02228_si_007.pdf [file nl4c02228_si_007.pdf]

Understanding the growth of electrodeposited PtNi nanoparticle films using correlated *in-situ* liquid cell transmission electron microscopy and synchrotron radiation

Magdalena Parlinska-Wojtan[1], Tomasz Roman Tarnawski[1], Joanna Depciuch[1,2,], Maria Letizia De Marco[3,1], Kamil Sobczak[4], Krzysztof Matlak[5], Mirosława Pawlyta[6], Robin E. Schäublin[7], See Wee Chee[3]*

[1] Institute of Nuclear Physics Polish Academy of Sciences, PL-31-342 Krakow, Poland

[2] Department of Biochemistry and Molecular Biology, Medical University of Lublin, Chodzki 1, 20-093 Lublin, Poland

[3] Department of Interface Science, Fritz-Haber-Institute of the Max-Planck Society, Faradayweg 4-6, 14195 Berlin, Germany

[4] Faculty of Chemistry, Biological and Chemical Research Centre, 02-089 Warszawa, Poland

[5] Solaris National Synchrotron Radiation Centre, Jagiellonian University, Czerwone Maki 98, 30-392, Krakow, Poland

[6] Silesian University of Technology, Materials Research Laboratory, Konarskiego 18A, 44-100 Gliwice, Poland

[7] ScopeM-Scientific Center for Optical and Electron Microscopy, ETH Zürich, 8093 Zürich, Switzerland

*Corresponding Author e-mail: magdalena.parlinska@ifj.edu.pl

Electrodeposition of PtNi films

PtNi nanoparticles have been fabricated by electrodeposition on glassy carbon electrode *ex-situ* and *in-situ* using LC-TEM. The reaction solution has been prepared using nickel(III) nitrate hexahydrate, M = 290.81 g/mol from Chempur and chloroplatinic acid hydrate, M = 2.43 g/ml, from Sigma Aldrich, according to the procedure described by Xiu et al. [1]. In a glass beaker, 0.43621 g of $Ni(NO_3)_2 \cdot 6H_2O$ has been solved in 15 ml of ultra-pure water and then 0.23359 g of $H_2PtCl_6 \cdot H_2O$ has been added. The reaction solution was next stirred at room temperature for 15 min. This solution will be further referred to as S1 solution. The working glassy carbon

---

[1] CURRENTLY AT: Institut de Physique et Chimie de Matériaux de Strasbourg, UMR 7504 CNRS, Université de Strasbourg, 23 rue du Loess, BP 43-67034 Strasbourg Cedex 2, France.

electrode has a cylindric shape, with a height of 8 mm and diameter of 5 mm. A platinum wire was used as a counter electrode and a silver chloride Ag/AgCl electrode was used as a reference electrode. For the electrodeposition reaction, cyclic voltammetry was performed, using SP-200 potentiostat from BioLogic. The electrodeposition parameters are shown in Table S1. The potential range was varied between E_1 and E_2, the scanning rate *dE/dt* was changed between 70 and 100 mV/s and the process was stopped after *n* = 5, 7, 10 or 15 potential cycles. Two Pt:Ni molar ratios were tested 25:75 and 35:65, respectively. After the deposition, the electrodes with the nanoparticle films were rinsed with water and dried at ambient atmosphere.

Table S1 Variation of PtNi electrodeposition parameters.

Parameter	Sample series									
	**A1**	**A2**	**A3**	**B1**	**B2**	**B3**	**C1**	**C2**	**D1**	**D2**
E_1 **[V]**	1.2			1.2			1.2		**1.2**	**0.8**
E_2 **[V]**	-0.8			-0.8			-0.8		-0.8	-0.8
dE/dt **[mV/s]**	100			**100**	**85**	**75**	85		85	80
n	**15**	**10**	**5**	7			7		7	7
Pt:Ni precursor ratio	35:65			35:65			**35:65**	**25:75**	35:65	35:65

For the first few *ex-situ* experiments, a wider potential range was used. Subsequently, we reduced the upper potential from 1.2 V to 0.8 V (Fig. S8). Limiting E_1 (the upper potential limit) to 0.8 V resulted in a higher nickel concentration in the nanostructure (green box in Table S3, Fig. S8), as the oxidation and dissolution of Ni is limited at this potential, whereas the platinum concentration remains high, since Pt is less liable, in general, to oxidation.

The surface morphology of the films fabricated *ex-situ* was observed using a scanning electron microscope (SEM) Tescan Vega 3 operating at 30 kV. The chemical composition was analyzed by EDS at 10 kV.

1.1. Number of cycles variation

In the first experiment, the electrodeposition was conducted from solution 1 with varying number of cycles, which allowed to reduce nanoparticles size. Fig. S1, Fig. S2 and Fig. S3 show respectively the voltammetry plots for those electrodeposition reactions, as well as SEM images of the surface of the electrodeposited films.

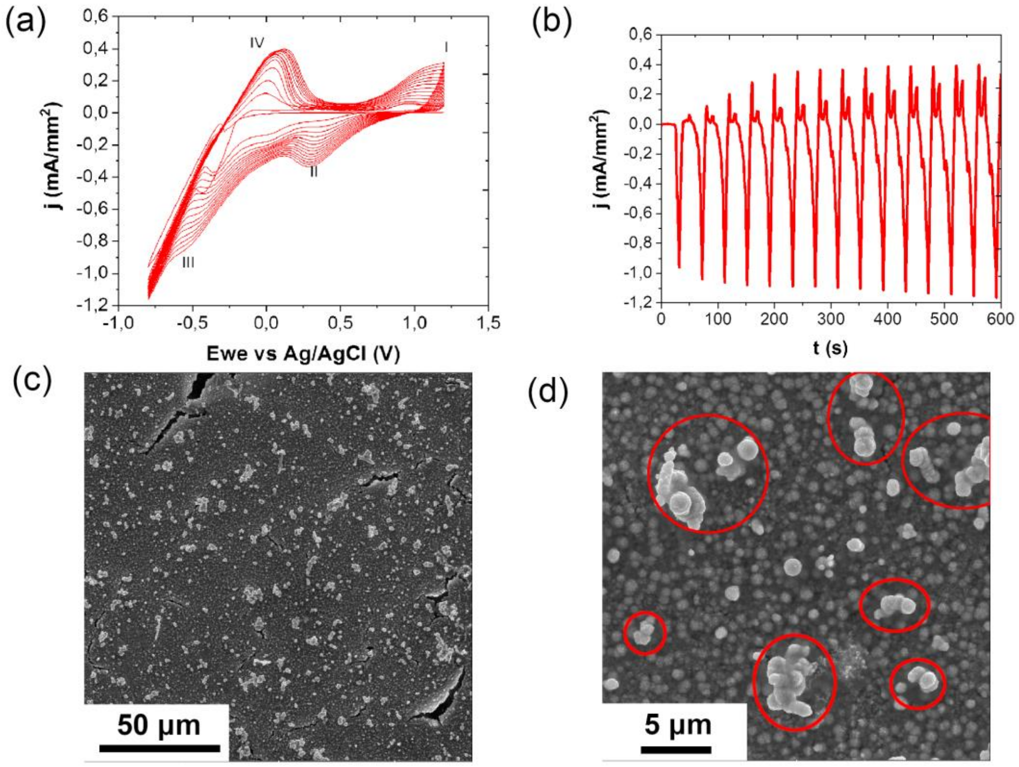

Fig. S1 Sample A1 a) Voltammogram with reduction peaks: (II) platinum, (III) nickel – shifted to ca. -0.65 V (literature value -0.59 V [1]). b) Current density vs time plot showing increasing currents at maximum and minimum potentials with every cycle – the nanoparticles film becomes thicker with every cycle, thus changing its conductivity [2]. The increase of the reduction peaks indicates also, that the electrodeposition does not cover the working electrode completely and there are still some active surface sites available. SEM images: c) overview showing bright chloride aggregates and multiple cracks; d) bright aggregates marked with red circles.

Fig.S1-S3 show voltammetry plots for electrodeposition samples A1, A2 and A3 using parameters from Table S1. There are reduction peaks on each of them, marked as II and III on the cathodic (bottom) curve and IV on the anodic (upper) curve. According to Xiu et al., II indicates platinum reduction., whereas III might indicate nickel reduction, although it seems to be slightly shifted – Xiu et al. report -0.59 V (Xiu *et al.*, 2015). To better understand the dynamics of that process and how those peaks evolve in time, time scale current density plots are also presented in Figures S1-S3. Here it is clear that the reduction peaks actually increase – the maxima and minima grow with each cycle. Moreover, the edge points of the plot – currents at maximum and minimum potentials – are higher with every cycle. This effect is correlated to the fact, that the nanoparticle film is naturally more thick with every cycle, so it changes the conductivity, and that is why the current increases (Yu *et al.*, 2017). The huge anodic peak at 0 V is most likely correlated with hydrogen redox reactions and its adsorption on platinum surface (Xiu *et al.*, 2015), (Ustarroz *et al.*, 2014).

The SEM imaging revealed that nanoparticles films fabricated with have numerous cracks, which are visible on Fig. S1(c) and Fig. S2(c). When *n* is low, it is possible to obtain nearly a monolayer of nanoparticles, whereas with high *n* value, nanoparticles create a multi-layer structure that tends to crack. Another undesirable effect observed for a higher numb er of cycles,

was the growth of aggregates on the surface. They are visible on Fig. S1(d) as numerous large brighter spots, marked with red circles. On the A2 and A3 samples, the aggregates were smaller and not so numerous, which can be seen on Fig. S2(d) and Fig. S3(d). Additionally, it is visible on Figure S3(c) that the surface is not so cracked as for the previous two samples. There are however big round black spots, in which there are no nanoparticles deposited or the film is extremely thin. Those spots were caused most probably by gas bubbles, which are created on the electrode during electrodeposition.

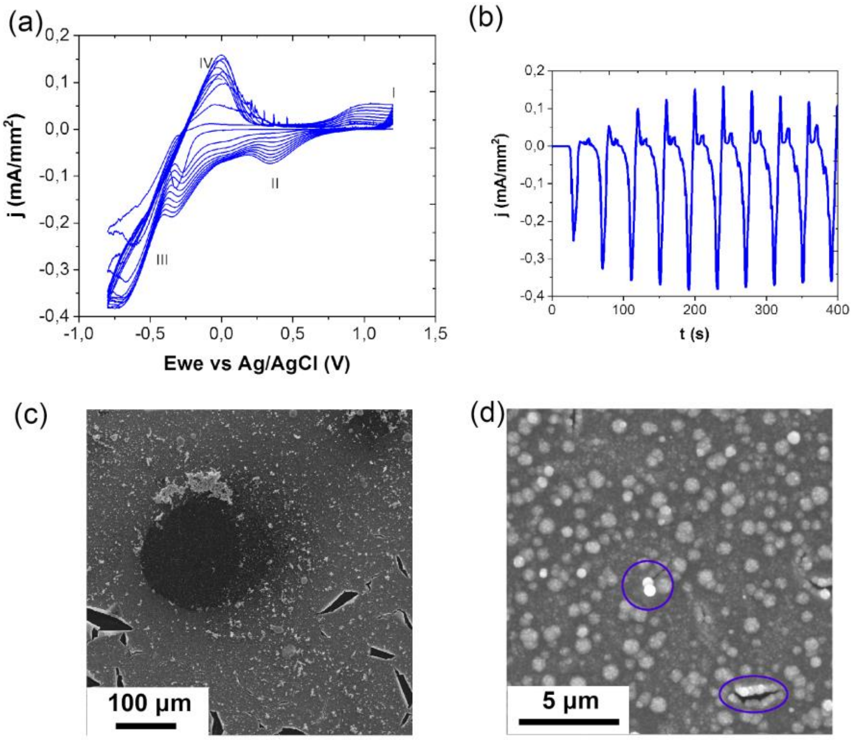

Fig. S2 **Sample A2** a) Voltammogram with reduction peaks: (II) platinum, (III) nickel – shifted to ca. -0.65 V; b) Current density vs time plot; SEM images: c) bright aggregates, black cracks and a black spot with almost no nanoparticles; d) bright aggregates marked with blue circles.

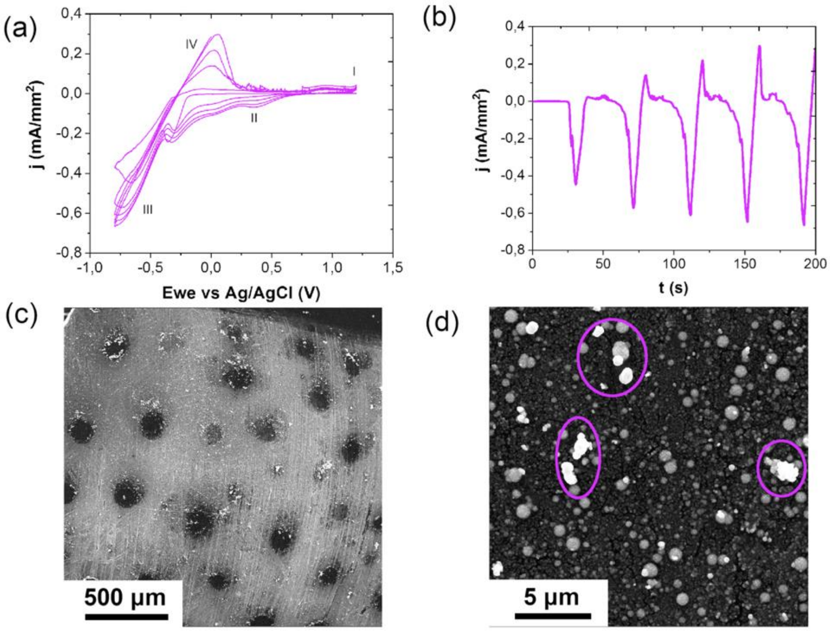

Fig. S3 **Sample A3** a) Voltammogram with reduction peaks: (II) platinum, (III) nickel – shifted to ca. -0.65 V; b) Current density vs time plot with reduction peaks increasing with each cycle; SEM images: c) for lower *n* value, less cracks are formed due to decreasing the film thickness, but numerous black spots are visible; d) bright aggregates marked with violet circles.

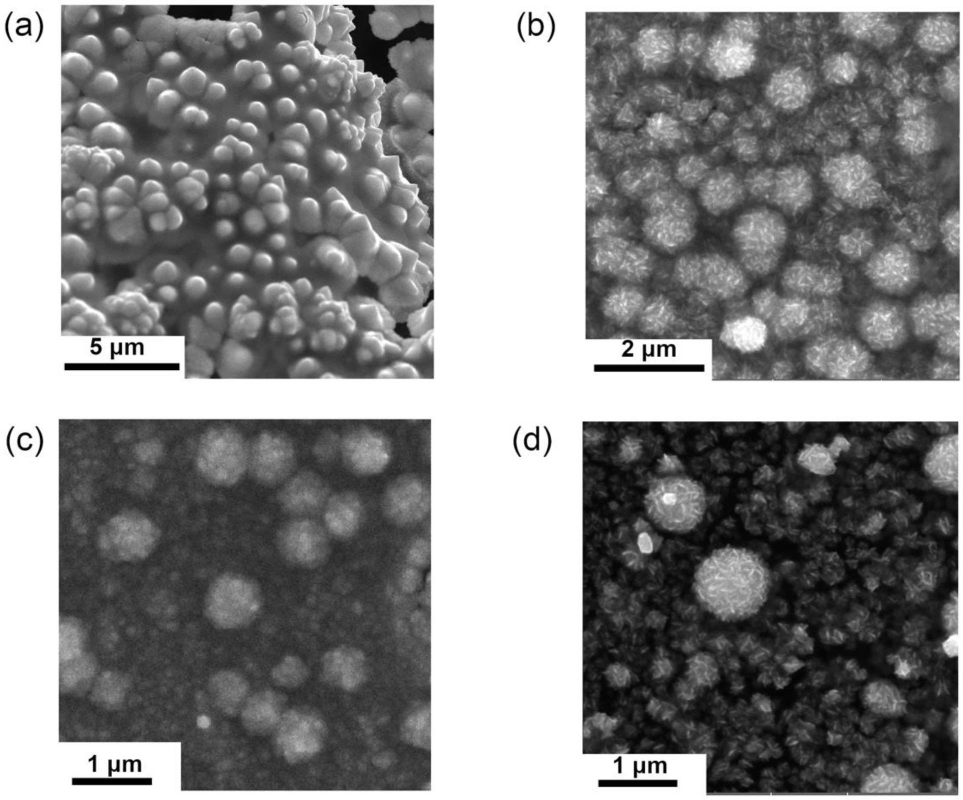

Fig. S4 SEM images of the nanoparticle films: a) **Sample A1**, magnification of large chloride aggregates – undesirable by-product of the electrochemical deposition. B) **Sample A1**, PtNi nanoparticles with spherical shape and highly porous surface, with a uniform diameter of ~1 µm; c) **Sample A2**, shows a large spread in the size distribution from several nanometers to individual spherical NPs with a diameter of ~500 nm; d) **Sample A3**, PtNi NPs with a diameter of 200-300 nm and only few spheres bigger than 500 nm are to be seen.

In Figure S4, a comparison of the structure of samples A1, A and A3 fabricated by varying only the number of deposition cycles is presented. As we can see, they consist of round shaped nanoparticles / agglomerates and have a highly porous surface structure, which is desired. The structure on Fig. S4(a) is a chloride aggregate and in next attempts we tried to minimise their amount in the films. Those are undesirable by-products and it is directly reported that their adsorption might inhibit metallic nanoparticles deposition (Ustarroz *et al.*, 2014). Therefore, it was crucial to reduce their deposition in next experiments.

1.2. Scanning rate variation

For B series, where the scanning rate was varied, also three experiments have been performed with the parameters presented in Table S1, using solution 1.

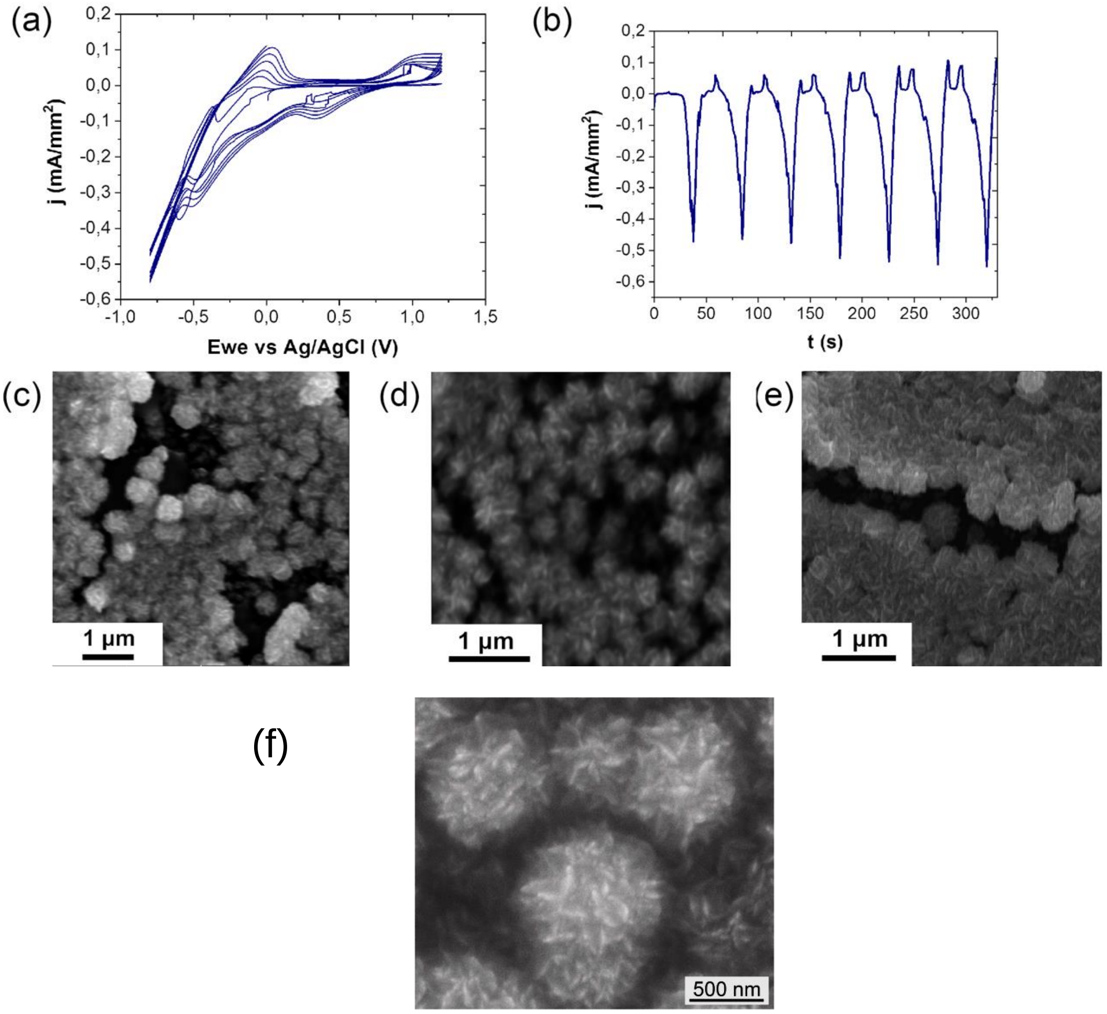

Fig. S5 **Sample B2**: a) Voltammogram with some minor fluctuations around 0.35 V and 1.0 V and noticeable reduction peaks. The hydrogen peak is lower than in Fig. S1-S3, which were made in higher scan rate. Decreasing the scanning rate reduces hydrogen evolution on the working electrode and leads to more stable metal deposition; b) Current density vs time plot with minimum and maximum current values growing in time with each cycle, as the nanoparticles film thickness increases. SEM images for samples: c) B1; d) B2 and e) B3, respectively. The nanoparticles are composed of spherical grains**,** with a diameter below 500 nm, consisting of structures resembling the shape of desert roses.

It can be seen in Fig. S5 that the most homogenous structure with no visible cracks was obtained for sample B2. It is also worth noting, that the nanostructure of the nanoparticles was very similar in all samples – their size is ca. 500 nm in diameter, their shape is spherical and multifacial. Their form reminds of a desert-rose, which corresponds perfectly with the experiment from the previous section. Furthermore, this lack of shape variation also corresponds with previous reports about metallic nanoparticles fabrication, which state that the time of electrodeposition is irrelevant for the nanostructures shape. Ustarroz et al. conducted platinum electrodeposition using different scanning rate and they reported that shape was unaffected (Ustarroz *et al.*, 2014). The cyclic voltammetry plot and current plot for B2 sample were also similar to the previous ones. The reduction peaks correspond with reduction peaks from 3.1, the cathodic current reaches similar value for minimum potential, as well as anodic current for maximum potential. And despite some minor fluctuations, which can be seen around 0,35 V and 1,0 V, the general trend is the same and it is again clearly visible in the current time evolution – the current value grows in time, as the nanoparticles film thickness increases. The most noticable difference is that hydrogen peaks are lower here and that seems to be an advantage; H_2 evolution reportedly affects metal nanoparticles synthesis and its adsorption on metal atoms may limit the nucleation process (Allongue and Souteyrand, 1990).

1.3. Precursor concentration variation

In the third set of experiments, the influence of platinum and nickel concentration in the reaction solution has been investigated. EDS spectra for B series showed that Pt:Ni atomic ratio is around 90:10 and an attempt has been made to increase the Ni concentration in the nanoparticle film. In a new solution (which will be referred to as solution 2), 0.50235 g of nickel nitrate and 0.16745 g chloroplatinic acid was used, solved in 15 ml of ultra-pure water. So the total amount of material is preserved, but the Pt:Ni precursor ratio has been changed from 35:65 to 25:75. The rest of the procedure was as described in the Materials and Methods for solution 1. Using solution 2, electrodeposition was performed two times, using the parameters listed in Table S1 for C series.

The voltammogram in Fig. S6 indicates that the platinum reduction peak on cathodic curve (around 0,35 V, as it was marked in Fig. S1a) was indeed lower in this experiment, as well as the anodic current for maximum potential. That should suggest poorer Pt synthesis on the working electrode. However, the EDS spectra did not show any significant difference, compared with the previous results.

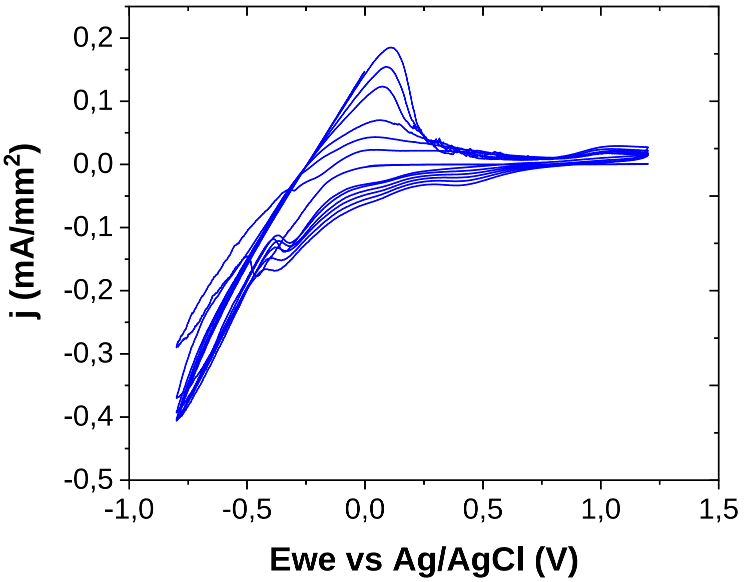

Fig. S6 Voltammogram for sample C1. The platinum reduction peak and the anodic current for maximum potential are lower than for sample series B, indicating poorer Pt synthesis.

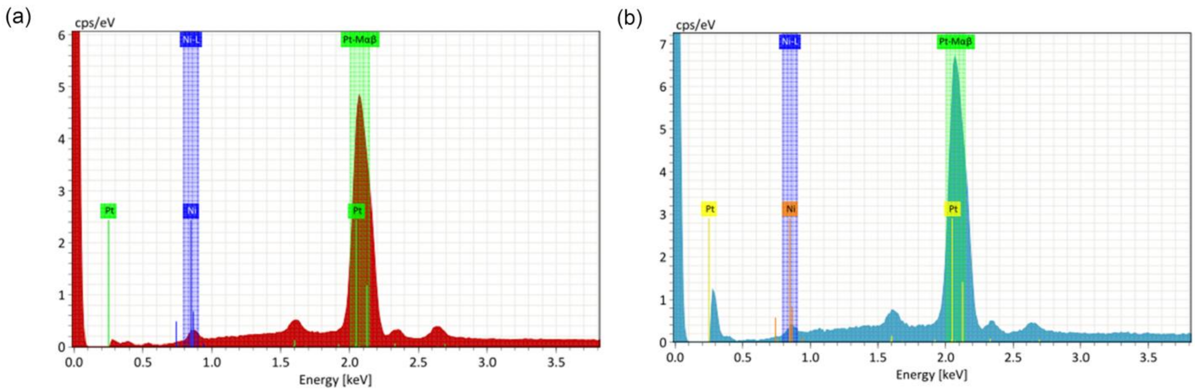

Fig. S7 EDS spectra for samples from (a) B series and (b) C series.

Table S2 Pt:Ni atomic ratio for samples from B series and C series. Despite a modification in the precursor concentration, the atomic ratio did not change significantly.

Sample series	Pt [%]	Ni [%]
B	90	10
C	91	9

1.4. Scanning range variation

To increase the nickel concentration in the nanoparticle films, the voltammetry scanning range has been limited, and electrodeposition has been conducted from solution 1 with parameters from Table S1 for D series. Consequently, platinum concentration in the nanoparticles decreased in favor of nickel.

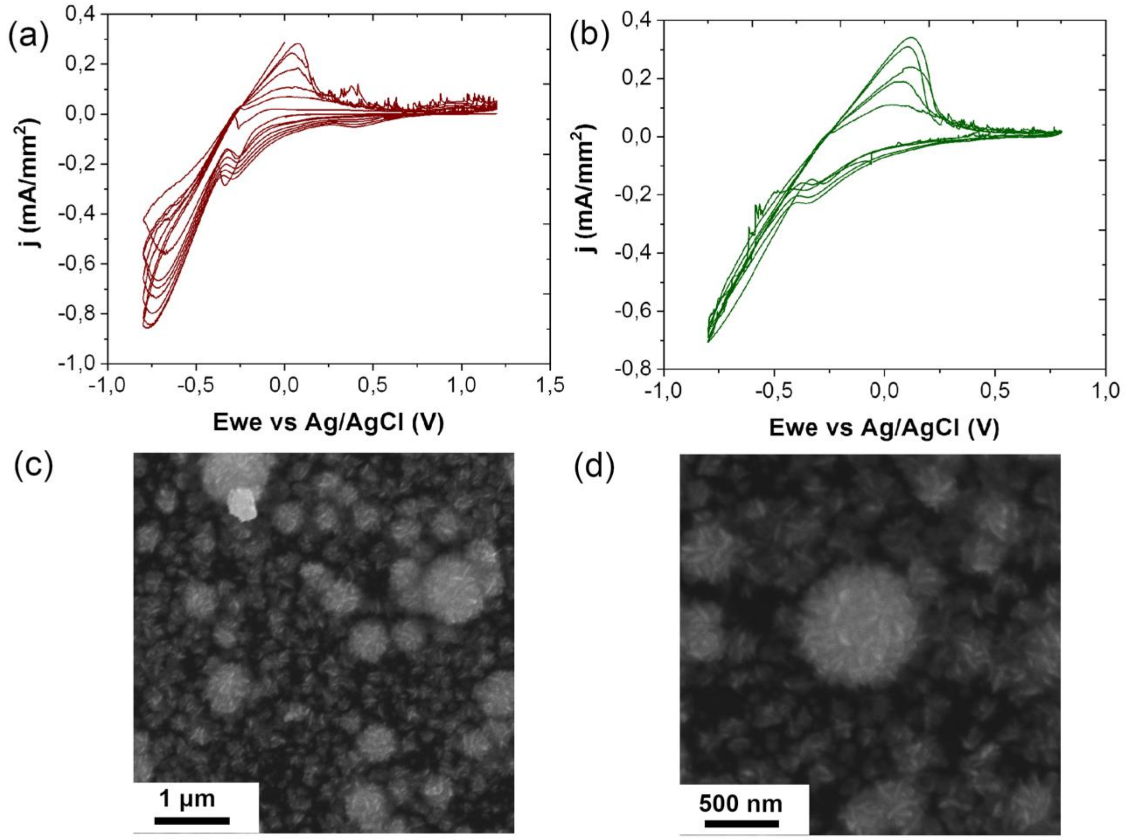

Fig. S8 Sample D series. Voltammetry plots of samples: a) D1 showing the reduction peak for platinum; b) D2 the Pt reduction peak disappeared, which is correlated to limited Pt deposition. c-d) SEM images of D2 sample. The NPs have the same structure as in A and B series, but are smaller. d) magnification of a large nanoparticle with 500 nm in diameter.

Since platinum reduces itself more easily, we can assume that platinum NPs deposit first on the electrode and nickel NPs deposit on their surface. It is also visible on voltammetry plots, that as the electrodeposition proceeds, we first observe platinum reduction. But when the potential range has been limited, the whole reaction has been altered. It is clear when we compare voltammograms of D1 and D2 sample (see Fig. S8A and S8B). The reduction peak for platinum is still visible in D1, for 0,35 V, but it disappears almost completely in D2. That means, platinum deposition has been reduced, whereas nickel deposition should remain unaffected, although the cathodic current is slightly lower in Fig. S8A than Fig. S8B. Also the variation of cathodic current is smaller for D2; it does not increase in time as it was observed in previous experiments. For D1, current density values for -0,8 V potential are higher with each cycle, indicating that electrodeposition speeds up (Yu *et al.*, 2017). But in D2 current is almost constant which suggests rather slow and stable growth of nickel nanoparticles. Decreasing the potential range reduces the dynamics of nickel NPs deposition and it takes more time to fabricate NPs film.

Overall, we can say that electrochemical data are consistent with EDS mapping – by limiting potential range, precursor reduction has been modified and platinum electrodeposition has been reduced, which is visible on the voltammograms and confirmed by EDS.

It is also worth to mention, that nanostructure of the nanoparticles remained the same as in the previous experiments, which can be seen on Fig. S8, although the size of particles is smaller, which also may be caused by slower growth. There are various particles with only a few hundred nanometres in diameter and only some of them have 500 nm.

Table S3 Pt:Ni atomic ratio for sample D1 and D2. After limiting the potential range, platinum concentration in the NPs decreased in favour of nickel.

Sample	Pt [%]	Ni [%]
D1	90	10
D2	85	15

1.5. *In-situ* LC-TEM electrodeposition of PtNi films with Poseidon holder

For the *in-situ* TEM imaging of the nanoparticle films growth, the Poseidon Select holder from Protochips was used (see Fig. S9). The liquid cell consists of two Si chips with a silicon nitride window. The top chip is equipped with a working carbon electrode (WE), located in the middle of the window, the counter electrode (CE) and the reference electrode (RE). When the holder is sealed, these electrodes are in contact with the three gold electrodes on the holder itself, thus the connection with the potentiostat can be established. All electrochemical processes were controlled by potentiostat (BioLogic SP200) and EC-Lab software. The most important parameters during real time TEM imaging are: the electron dose rate (*EDR*) and the solution flow rate through the liquid cell (*dV/dt*). The parameters for electrodeposition in the liquid cell are as listed in for sample D2:

Table S4 Parameters for the *in-situ* TEM electrodeposition of PtNi nanoparticles

E_1 [V]	E_2 [V]	*dE/dt* [mV/s]	*n*	*EDR* [e/Å^{2}s]	*dV/dt* [µl/min]
**0.8**	**-0.8**	**80**	**7**	**0.04**	**3**

High resolution STEM imaging, as well as *in-situ* TEM experiment were performed in a FEI Titan 80-300 operating at 300 kV, equipped with a FEG cathode and a Cs corrector.

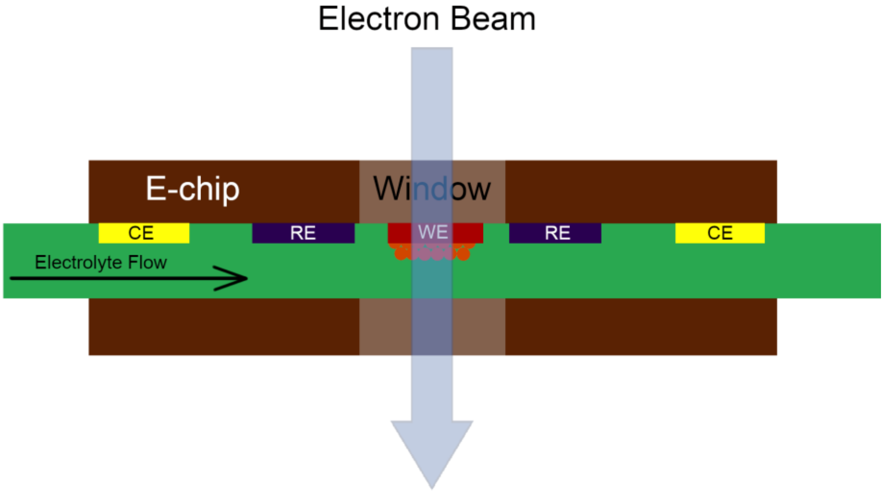

Fig. S9 Cross-section schema of the Poseidon liquid cell. The cell is composed of two E-chips with a silicon nitride window. CE – Counter Electrode, RE – Reference Electrode, WE – Working Electrode. The electrolyte flows through the cell and the nanoparticles (orange) are deposited on the WE.

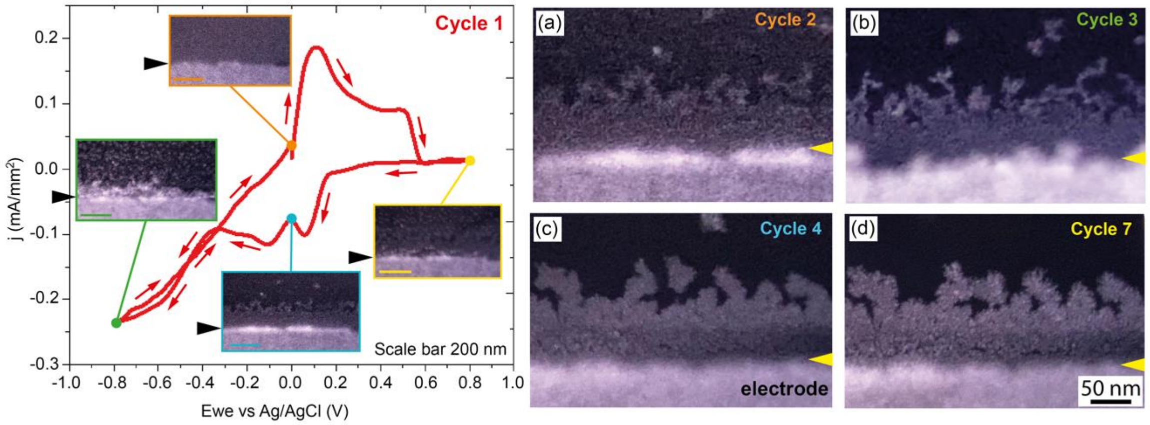

Fig. S10 Left image: Cyclic voltammetry curve illustrated by real-time HAADF TEM images of the first cycle of PtNi NPs film growth on the electrode inside the liquid cell. a) – d) HAADF STEM time frames of the PtNi NPs growth on the electrode inside the liquid cell. The branched structures formed in cycle 2 grow in thickness with every next cycle: a) Cycle 2; b) Cycle 3; c) Cycle 4; d) Cycle 7. The black and yellow triangles show the electrode edge, which is at the bottom of the images.

The electron dose rate was 0.4 e/Å^{2}s. The first four electrodeposition cycles are shown in Movie 1. Real-time imaging allowed correlating the obtained images with voltammetry data to analyze the process dynamics. The 1st cycle of the electrodeposition process with the voltammogram illustrated by the LC-TEM images is presented in Fig. S10 (left). Images captured at different times during the first CV show, that nanoparticle growth starts immediately after the potential has been applied. After about 10 seconds, when the potential reaches the maximum value of 0.8, a dense nanostructured film of NPs is formed on the electrode. In the following 30 seconds, fast particles deposition is observed.

Fig. S10(a-d) shows time frames from the next cycles of the electrodeposition. Fig. S11 shows a magnification of an aggregate attachment to the film. After four cycles, no net growth is observed anymore, and only particle coarsening is observed. This can be explained by hindered

transport of metal ions caused by a growing nanoparticle layer on the electrode. When the layer gets too thick, ion diffusion reduces, and the electrodeposition process stops [2].

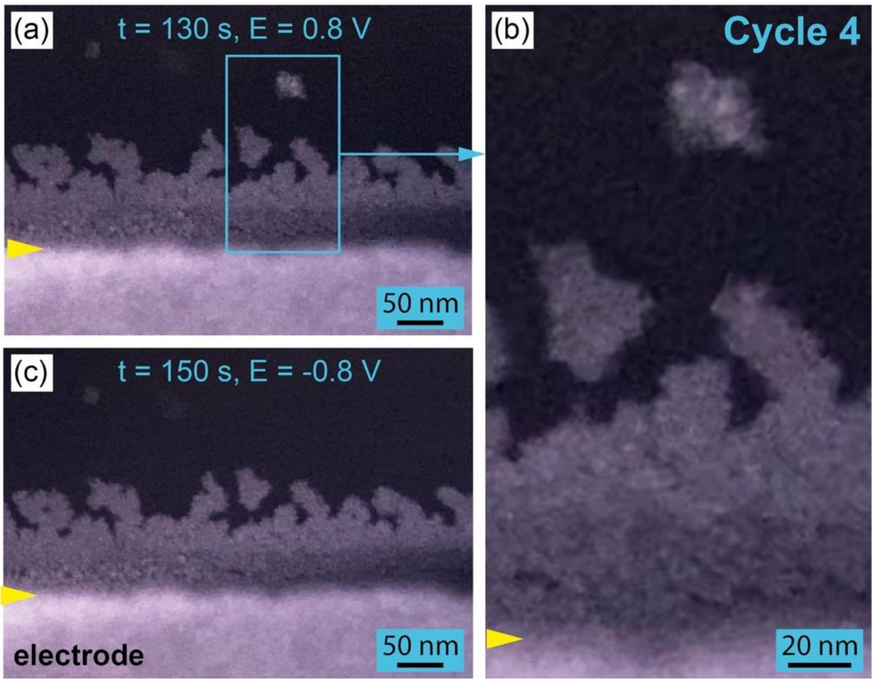

Fig. S11 HAADF TEM time frames of the PtNi NPs growth on the electrode inside the liquid cell. a) Cycle 4, max. voltage; b) magnified view of the PtNi film with an agglomerate floating towards the electrode; c) Cycle 4, min. voltage. The yellow triangles show the electrode edge, which is at the bottom of the images.

The electron beam-induced growth of a PtNi particle from the liquid phase and its attachment to the electrodeposited layer are presented in Movie 6. The beam effect in promoting particle nucleation is also visible in Fig. S12c. The right arrow indicates the area scanned by the electron beam for 30 seconds, which is full of aggregates visible above the electrode edge. The left arrow indicates a fresh, non-illuminated area of the film-coated electrode, without any floating clusters.

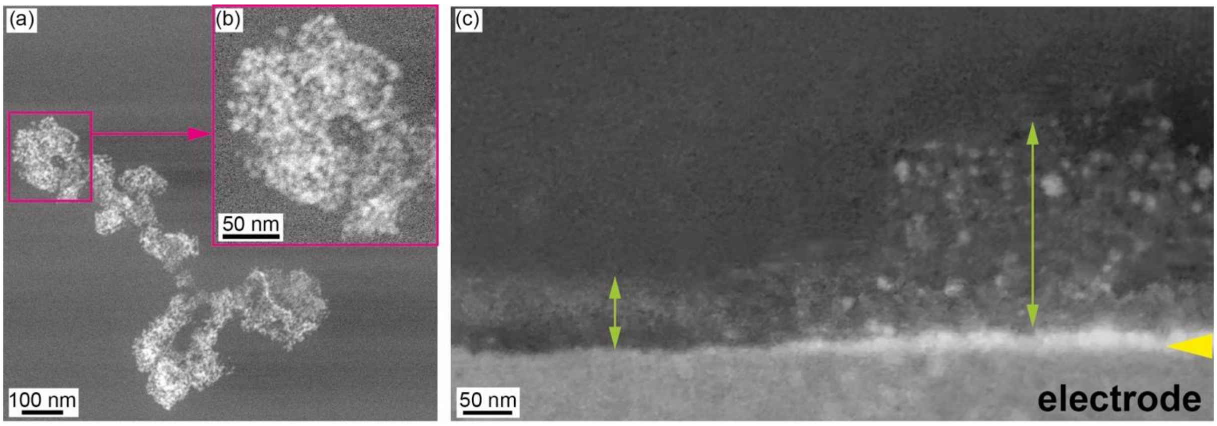

Fig. S12 (a) HAADF STEM image of freely floating PtNi nanoparticle in the liquid cell; (b) magnification of the structure marked in the (a) image The electrode edge is at the bottom below the image, the NPs float in the liquid; (c) HAADF STEM image of the electrode edge with the electrodeposited PtNi nanograined film. Comparison between the structure stimulated (right arrow) and non-stimulated (left arrow) by the electron beam. The electrode edge is indicated with the yellow triangle in image (c).

After finishing the voltammetry cycling, the flow was still maintained and the liquid cell was observed, in order to analyse the grown nanostructures and examine, if they evolve under the electron beam itself. No particle degradation has been observed, unless an EDS point measurement was performed. In Fig. S13, an aggregate is presented before (a) and after (b) it was analysed by EDS and the nanoparticle was destroyed by the electron beam. This process is also presented in Movie 5. The obtained EDS spectrum is presented in Fig. S13c.

It is widely discussed in electrochemical experiments whether electrodeposition starts with nanoparticles growing at the electrode, or if clusters of nanoparticles form in the liquid and then diffuse to the electrode's edge. Without real time imaging, it would be impossible to distinguish between these two possibilities. From the frames in Figure S10, it can be concluded that the film was growing directly on the electrode in the first stage of electrodeposition. It took ca. 30 seconds before the first clusters started to appear above the edge. However, the clusters were appearing only in the region scanned by the electron beam, as their formation was a beam-induced effect. It must be kept in mind, that high voltage in the TEM may enhance the synthesis process and change its dynamics, although the scale of that effect is not always clear [3]. The electron beam may cause radiolysis, atomic replacements, gas bubbles [4], [5] pH fluctuations, and uncontrolled temperature increases of the solution inside the liquid cell [6], [7], [8]. It has been reported, that radiolysis induced by the beam generates aqueous electrons, e_{aq}^{-}, which may act as strong reducing agents. Some researchers reported, that such aqueous electrons are actually able to reduce metal ions from the precursor, forming metal clusters and eventually nanoparticles [9], [10]. This effect has been observed here. It can be concluded from the Movie 1, that the clusters appear and grow intensively above the electrode edge in the region scanned by the electron beam during cycle 1 and 2. However, when the sample was moved to a new region, not illuminated by the electron beam, no such effect was observed, Fig. S12(c). From these observations we can conclude, that the PtNi nanofilm grows directly on the working electrode, while the effect of nanoparticles forming in above it is induced by the electron beam.

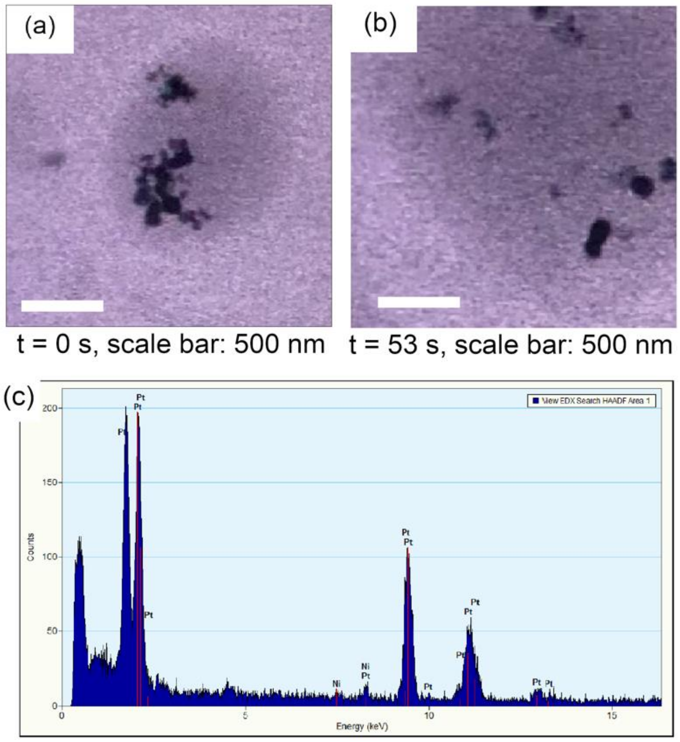

Fig. S13 BF STEM images of PtNi NPs floating in the liquid cell: (a) before EDS acquisition; (b) after EDS acquisition; (c) obtained EDS spectrum of the NPs depicted in (a) & (b) (Movie 5). The images were taken in the liquid on floating particles.

1.7 *In-situ* LC-TEM electrodeposition of PtNi films with Hummingbird holder

Additional *in-situ* LC-TEM experiments were carried out with a Hummingbird holder equipped with a bulk Ag/AgCl reference electrode (3M), which allows to compare *ex-situ* with *in-situ* data. The hummingbird holder was connected with a BioLogic SP200 potentiostat, used to cycle the potential between 0.8 V and -0.8 V *vs* Ag/AgCl with varying scanning rate. The measurements were carried out in a Thermofisher Titan (S)TEM, with an acceleration voltage of 300 kV. All the experiments were carried out at a magnification 20 kX with an electron dose was kept at 4.56 electrons nm^{-1} s^{-1}.

1.8 *In-situ* STXM experiments at the synchrotron

The XANES measurements were performed at the STXM-branch on the DEMETER beamline at the National Synchrotron Radiation Centre SOLARIS. The STXM end-station, designed by PhD Tolek Tyliszczak, is equipped with a commercial ElectroChemistry Cell (ECC) from Hummingbird Scientific, which was designed for this microscope and synchrotron studies using soft X-rays. This ECC system can work in stationary environment or, thanks to the syringe pump used, in a given fluid flow (gases or liquids). Standard electrochemistry chips have three

platinum micro-electrodes: working, reference and counter electrode (area factor 2:1:4). All electrochemical processes were controlled by potentiostat (BioLogic SP200) and EC-Lab software. SOLARIS STXM end-station is a typical scanning microscope, in which the sample placed in the focus of the Fresnell Zone Plate (FZP) is moved by precise XY piezo scanner. To ensure the repeatability of the required precision of positioning the sample relative to the FZP, a two-channel interferometric system with differential sensor heads was used. The photomultiplier tube (PMT) with a P-43 scintillator was used to detect X-ray intensity passed through the sample. The transmission signal collected in STXM is converted into the optical density (*OD*), which is sensitive to the sample density or thickness, and the composition (interest chemical element).

$OD = -ln(I / I0)$, where I is the intensity in a ROI (region of interest), $I0$ is the intensity of non-sample place (background).

Before the measurements the monochromator was calibrated using absorption in N2 gas. From the collected data, it was estimated that the differences for N_2 peak before and after experiment was 20 meV. Consequently, for Ni, changes in the peak position were +/- 80 meV.

Axis2000 software was utilized to align the STXM image stacks and extract Ni XANES spectra from them [11].

1.9 *Post-mortem* (S)TEM and EDS analysis of the electrodeposited PtNi films

The *post-mortem* analysis of the PtNi films was performed using several transmission electron microscopes. The PtNi film grown on the electrode of the large chip in the LC-TEM experiment using the Poseidon holder was analysed using a JEOL JEM-ARM300F Grand ARM "Vortex" operating at 300 kV. The post-mortem analysis of the PtNi films grown in the STXM experiment and the LC-TEM Hummingbird experiment was done in a Thermofisher Titan (S)TEM, with an acceleration voltage of 300 kV.

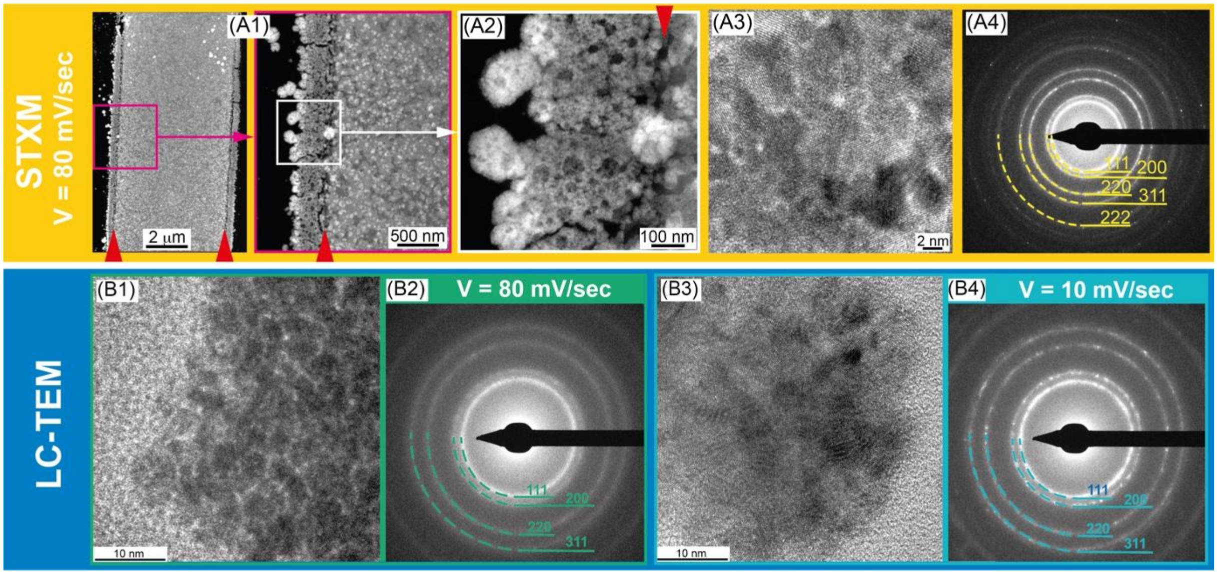

Figure S14 *Post-mortem* (S)TEM analysis of the PtNi films with the corresponding selected area diffraction patterns, grown in the: (A) synchrotron using STXM technique; (B) LC-TEM using the Hummingbird holder conducted at different cycling speeds. The red triangles indicate the electrode edge. See text for details.

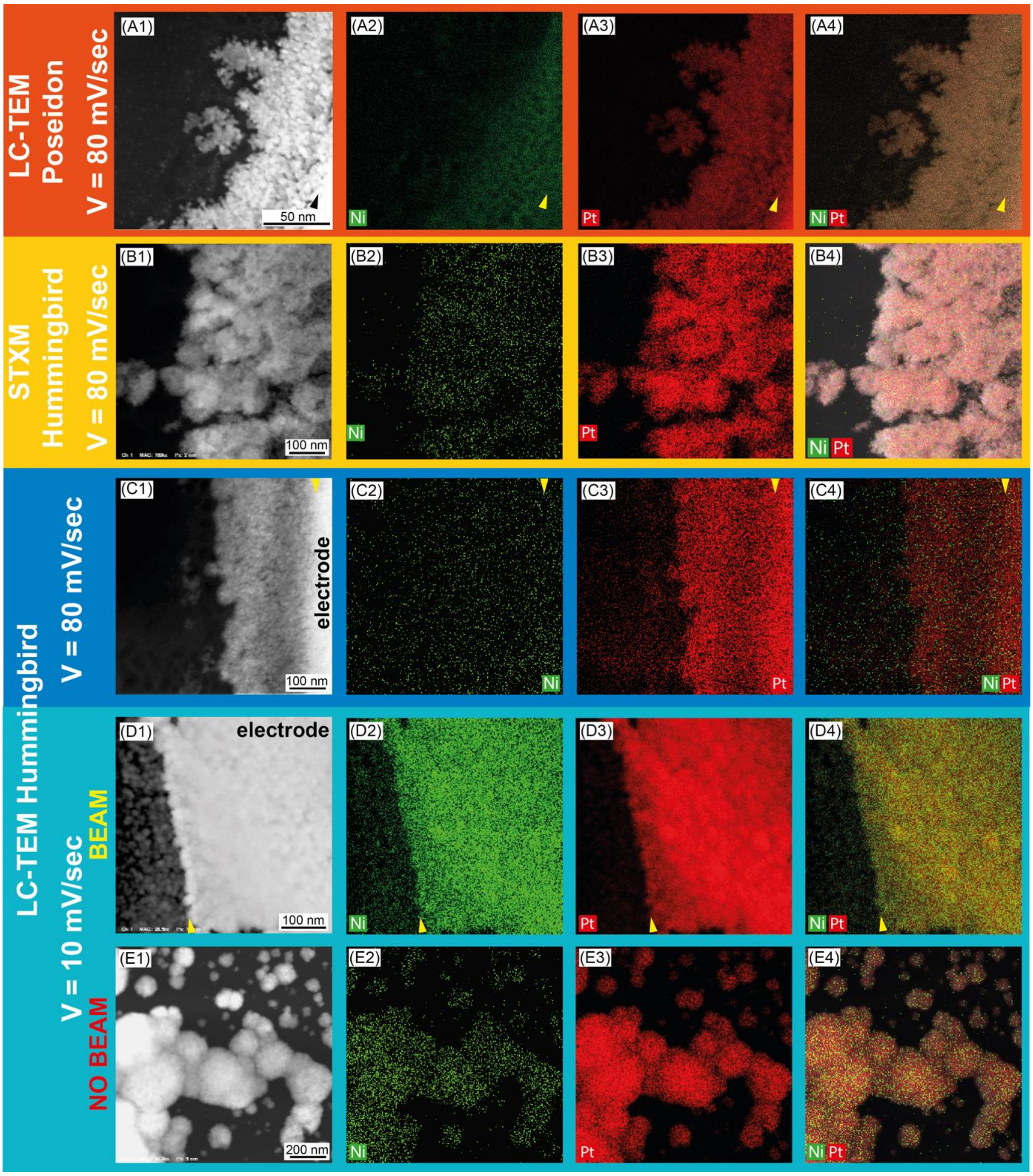

Fig. S15 Comparison of *post-mortem* EDS mappings of all three experiments: (1 – orange frame*) in-situ* LC-TEM experiments using the Poseidon holder; *in-situ* STXM (2 – yellow frame); *in-situ* LC-TEM Hummingbird experiments conducted at two different cycling velocities: V = 80 mV/sec (3 – turquoise frame) and V = 10 mV/sec (blue frame) in an (4) area illuminated with the electron beam and (5) without the electron beam. Each column represents respectively: (a) HAADF image of the electrode; (b) Ni map; (c) Pt map; (d) overlayed Ni + Pt maps. The yellow triangles indicate the electrode edge. For the STXM experiment, the electrode edge is always at the right edge of the map, while for the E line, the electrode image is outside of the image frame.

Post mortem EDS mappings of the PtNi film electrodeposited *in-situ* are presented in Fig. S15. The maps confirm that the film is composed of a uniform alloy of Pt and Ni with small concentration of Ni independently on the technique, holder and cycling speed. The higher intensity of the Ni signal for the sample in Figure S15 D-line can be related to two different causes. First, in this area we observed an important beam effect which accelerated the deposition of both Pt and Ni, producing a thicker and more compact layer compared to non-irradiated areas (see Fig. S15 line E). Moreover, the slower scanning rate in Fig. S15 D and E might also cause a higher percentage of Ni to depose.

Table S5: Table showing the respective concentrations of Pt and Ni in the PtNi film depicted in Fig. S15 B1, grown in the *in-situ* STXM Hummingbird experiment conducted at V = 80 mV/sec. The last column corresponds to the average concentration of Pt and Ni from the entire film.

**Nº**	1	2	3	4	5	6	Map
**Pt at.%**	83	100	77	100	100	88	81
**Ni at.%**	17	0	23	0	0	12	19

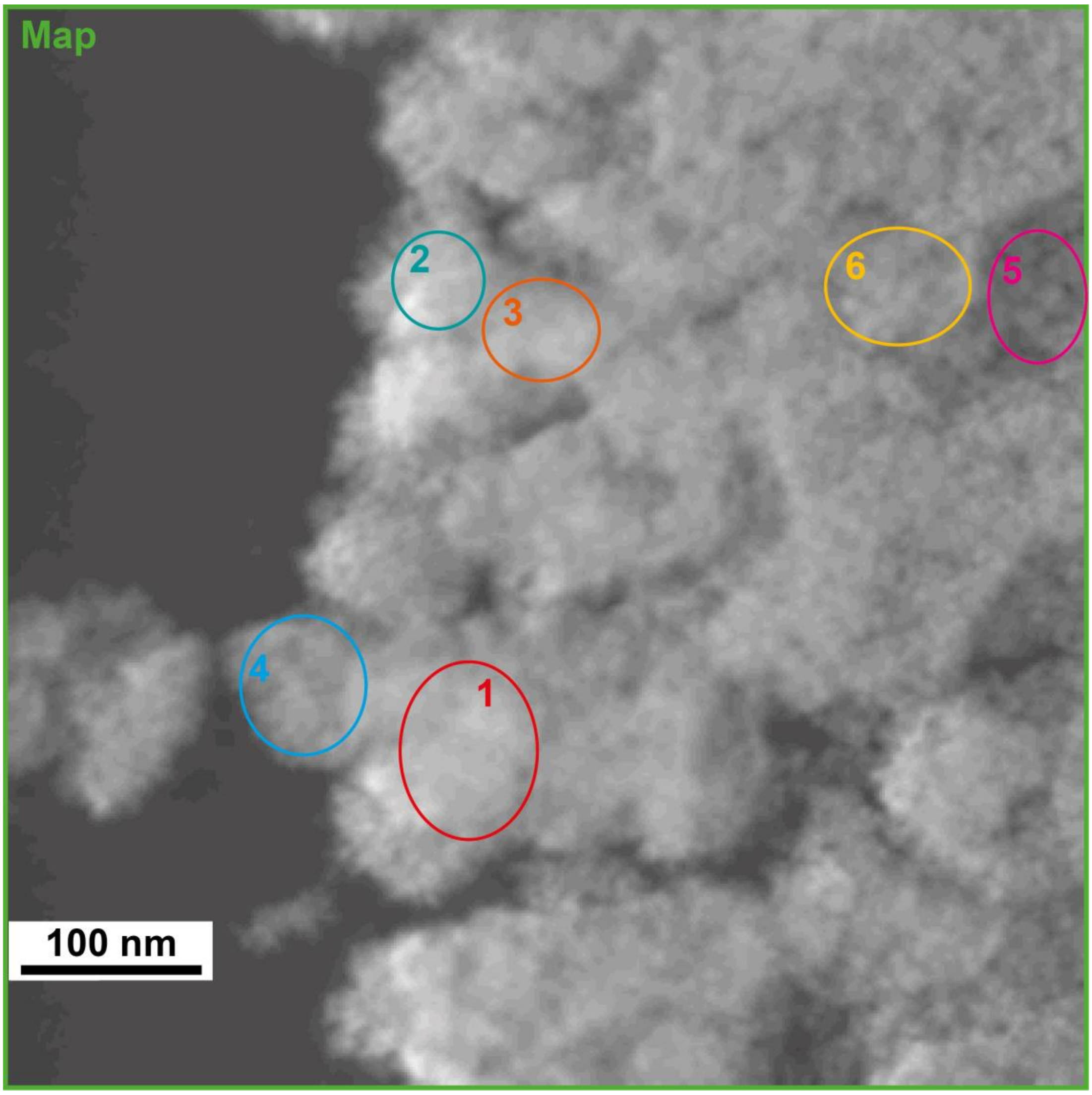

Fig. S16 HAADF image of the PtNi film depicted in Fig. S15 B1, grown in the *in-situ* STXM Hummingbird experiment conducted at V = 80 mV/sec.

Local variations of concentrations of Pt in the grown films were observed, independently on the experiment. Fig. S16 and S17 show the HAADF STEM images of the electrodeposited PtNi layers from Fig. S15 B1 and C1 with marked areas, from which EDS evaluation of the Pt and Ni concentrations was performed. The atomic concentrations are presented in Table S5 and S6, respectively.

Table S6: Table showing the respective concentrations of Pt and Ni in the PtNi film depicted in Fig. S15 C1, grown in the LC-TEM hummingbird experiment conducted at V = 80 mV/sec. The last column corresponds to the average concentration of Pt and Ni from the entire film.

**Nº**	1	2	3	4	5	6	7	8	Map
**Pt at.%**	81	83	100	96	91	98	97	100	81
**Ni at.%**	19	17	0	4	9	2	3	0	19

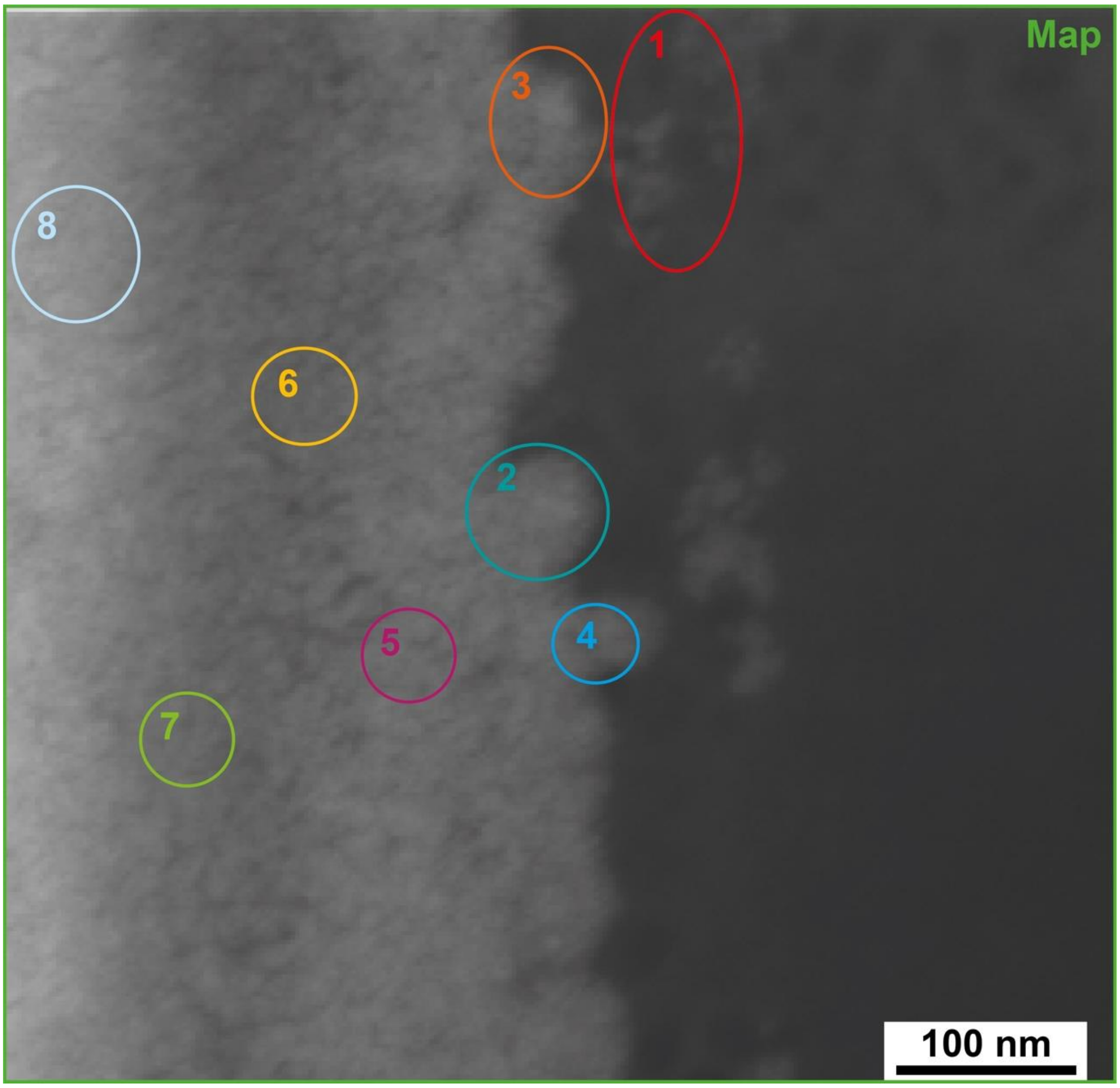

Fig. S17 STEM HAADF image of the PtNi film depicted in Fig. S15 C1, grown in the LC-TEM hummingbird experiment conducted at V = 80 mV/sec.

REFERENCES

[1] R. Xiu, F. Zhang, Z. Wang, M. Yang, J. Xia, R. Gui i Y. Xia, „Electrodeposition of PtNi bimetallic nanoparticles on three-dimensional graphene for highly efficient methanol oxidation,” *RSC advances,* tom 5, nr 105, pp. 86578-86583, 2015.

[2] Y. Yu, L. Sun, H. Ge, G. Wei i L. Jiang, „Study on electrochemistry and nucleation process of nickel electrodeposition,” *Int. J. Electrochem. Sci.,* tom 12, pp. 485-495, 2017.

[3] P. J. Herley i W. Jones, „Transmission electron microscopy of beam-sensitive metal hydrides,” *Zeitschrift für Physikalische Chemie,* tom 147, nr 1-2, pp. 147-159, 1986.

[4] R. F. Egerton, P. Li i M. Malac, „Radiation damage in the TEM and SEM,” *Micron,* tom 35, nr 6, pp. 399-409, 2004.

[5] J. M. Grogan, N. M. Schneider, F. M. Ross i H. H. Bau, „Bubble and pattern formation in liquid induced by an electron beam,” *Nano letters,* tom 14, nr 1, pp. 359-364, 2014.

[6] J. Cazaux, „Correlations between ionization radiation damage and charging effects in transmission electron microscopy,” *Ultramicroscopy,* tom 60, nr 3, pp. 411-425, 1995.

[7] B. C. Garrett, D. A. Dixon, D. M. Camaioni, D. M. Chipman, M. A. Johnson, C. D. Jonah, G. Kimmel, J. Miller, T. Rescigno, P. Rossky, S. Xantheas, S. Colson, ... i T. S. Zwier, „Role of water in electron-initiated processes and radical chemistry: Issues and scientific advances,” *Chemical reviews,* tom 105, nr 1, pp. 355-390, 2004.

[8] J. Yang, C. M. Andrei, G. A. Botton i L. Soleymani, „In liquid observation and quantification of nucleation and growth of gold nanostructures using in situ transmission electron microscopy,” *The Journal of Physical Chemistry C,* tom 121, nr 13, pp. 7435-7441, 2017.

[9] J. Belloni, M. Mostafavi, H. Remita, J. L. Marignier i M. O. Delcourt, „Radiation-induced synthesis of mono-and multi-metallic clusters and nanocolloids,” *New Journal of Chemistry,* tom 22, nr 11, pp. 1239-1255, 1998.

[10] R. G. Weiner, D. P. Chen, R. R. Unocic i S. E. Skrabalak, „Impact of Membrane-Induced Particle Immobilization on Seeded Growth Monitored by In Situ Liquid Scanning Transmission Electron Microscopy,” *Small,* tom 12, nr 20, pp. 2701-2706, 2016.

[11] A. P. Hitchcock, „aXis 2000 - Analysis of X-ray Images and Spectra,” [Online]. Available: http://unicorn.mcmaster.ca/aXis2000.html. [Data uzyskania dostępu: 29 February 2024].
